# Gap balancing improve squat function and knee function: a randomized controlled trial comparing gap balancing and measured resection

**DOI:** 10.1186/s13018-021-02367-9

**Published:** 2021-04-08

**Authors:** Qingfang Xiao, Bo Liu, Binghao Zhao

**Affiliations:** Department of Osteoarthrosis, Renmin Hospital, Hubei University of Medicine, No.39 Middle Chaoyang Road, Maojian District, Shiyan, Hubei China

**Keywords:** Gap balancing, Measured resection, Total knee arthroplasty, Knee osteoarthritis

## Abstract

**Objective:**

We compared the measured resection (MR) technique and the gap balancing (GB) technique in patients with knee osteoarthritis after primary total knee arthroplasty (TKA) in China to understand the effects of the two techniques on knee function and squat function.

**Methods:**

From March 2017 to September 2019, a prospective randomized controlled trial was conducted with 96 patients with knee osteoarthritis undergoing primary TKA from March 2017 to September 2019 randomized to GB group (*n* = 48) and MR group (*n* = 48). Intraoperative indicators (operation time, osteotomy volume of medial and lateral of posterior femoral condyles, external rotation angle) were recorded during operation. At 1, 3, 6, and 12 months after surgery, all the patients came to the hospital for review and underwent the pain severity, Western Ontario and McMaster University Osteoarthritis Index (WOMAC), knee joint range of motion, Oxford Knee Score (OKS), and American Knee Society Score (AKSS) tests. All patients were followed up for more than 1 year.

**Results:**

The osteotomy volume of the medial femoral condyle in the GB group was higher than that in the MR group (*P*<0.05), and the operation time in the GB group was shorter than that in the MR group (*P*<0.05). At 1, 3, 6, and 12 months after surgery, the pain severity in the GB group was lower than that in the MR group (*P*<0.05), the knee range of motion in the GB group was larger than that in the MR group (*P*<0.05), the WOMAC of the GB group was lower than that of the MR group (*P*<0.05), the OKS of the GB group was higher than that of the MR group (*P*<0.05), the AKSS of the GB group was higher than that of the MR group. The incidence of postoperative complications in the GB group (4.17%) was significantly lower than that in the MR group (18.75%) (*P*<0.05).

**Conclusion:**

The GB technique can effectively shorten the operation time, relieve pain, improve knee range of motion, improve squat function and knee function, reduce osteoarthritis index, and reduce the occurrence of complications, which is worthy of clinical popularization and application.

## Introduction

In recent years, with the increasing aging of population and obesity, the incidence of knee osteoarthritis has been increasing year by year [1]. In clinic, the course of knee osteoarthritis is long, and patients with knee osteoarthritis often have limited knee flexion activity, squat difficultly, and even cannot squat in serious cases. Among patients with knee osteoarthritis who received surgical treatment, total knee arthroplasty (TKA) yields high satisfaction and has been strongly recommended by the American Academy of Orthopaedics as a means of treatment for end-stage knee osteoarthritis [2, 3]. It can effectively alleviate the degree of knee deformity, provide pain relief, restore knee joint function, and help patients maintain an independent lifestyle. Currently, there are two different methods to achieve a balanced knee in TKA: measured resection (MR) technique and the gap balancing (GB) technique, or balanced resection [4]. As different osteotomy methods have different mechanical effects, their curative effects and prognosis are different. Whether gap balancing performs better than the measured resection technique has created strong controversy [5]. MR is the conventional TKA technique that relies on a reference line to set femoral rotation [6]. However, if the positioning is inaccurate, it is difficult to maintain the stability of the surgical joint line, which easily leads to poor rotation of the femoral prosthesis [7]. The alternative technique is the gap balancing which relies on optimal soft-tissue tensioning before the final femoral resection to achieve equal and balanced flexion and extension gaps [8]. However, the GB technique tends to elevate the joint line, which may affect the postoperative effect and safety [9].

Currently, both the GB technique and MR technique can be performed during TKA, however, there is a lack of randomized controlled trials (RCT) directly comparing these two techniques in patients with knee osteoarthritis in China. In this study, we conducted an RCT comparing the MR technique and GB technique in patients with knee osteoarthritis after primary TKA in China to understand the effects of the two techniques on knee function and squat function. Our hypothesis for this study was that the GB technique would have better functional scores and quality of life outcomes when compared with the MR technique in patients up to 1 year post-surgery.

## Material and methods

### Study design

A prospective, blinded randomized controlled trial was performed in our hospital. This study was approved by the Ethics Committee of our hospital and was conducted following the ethical standards of the Helsinki Declaration of 1975, as revised in 2000. All patients signed the informed consent.

### Patients

From March 2017 to September 2019, subjects with knee osteoarthritis treated in our hospital were enrolled in this study. The inclusion criterion included (1) degenerative knee osteoarthritis with Kellgren-Lawrence grade III or above, (2) treated conservatively for more than 3 months, (3) received primary TKA, (4) good patellar mobility and varus deformity of knee < 20 degrees, (5) no obvious systemic disease, normal heart, liver, kidney, and other important organ functions, and (6) complete clinical data. The exclusion criteria include (1) with a history of knee surgery or congenital deformity of knee joint;( 2) severe neurological lesions; (3) with contraindications to surgery; (4) allergic to analgesia and anesthesia; (5) patients taking long-term sleeping pills; (6) patients with simultaneous lesions of both knees; (7) with traumatic, rheumatoid, or secondary osteoarthritis; and (8) with obvious bone defects requiring pads during surgery.

### Sample size

Prior to this study, we conducted a power analysis. The sample size was calculated according to the previous formula [10]: *n*=(μ_1-α/2_+μ_1-β_)^2^s^2^(1+1/k)/(μ_t_−μ_c_)^2^. To achieve an α of 0.8 and a β of 0.80, would require a minimum of 36 patients in each group. And according to the provisions of the State Food and drug administration, 15% is taken as the release rate, so the sample size of this study is determined as *n* = 36 × 1/(1~0.15) = 47.88≈48 in each group.

### Interventions

#### Pain prevention regimens

All patients received pain prevention regimens as follows: All patients underwent femoral nerve block under conventional ultrasound guidance before the operation, and 1.5 g of Volexin was injected intravenously 30 min before operation (manufacturer: Suzhou Sino-chem Pharmaceutical Industry Co., Ltd.; approval number: GYZZ H19990367), 100 mg ropivacaine (manufacturer: Hebei Yi Pin Pharmaceutical Co., Ltd; approval number: GYZZ H20113463), 10 mg morphine (manufacturer: Northeast Pharmaceutical Group Shenyang First Pharmaceutical Co., Ltd; approval number: GYZZ H20121995), 1 ml De bao song (manufacturer: Hangzhou Mer sha dong Pharmaceutical Co., Ltd; approval number: GYZZ J20140160), and 0.5 ml epinephrine (manufacturer: Changchun Da Zheng Pharmaceutical Technology Co., Ltd; approval number: GYZZ H22022429) were mixed and diluted to 100 ml, and 95 ml was injected into the ligaments around the knee joint, anterior knee joint capsule, popliteal fossa tissue. Another 5 ml was injected into the soft tissue under the incision after the prosthesis was installed.

#### Surgical procedure

The patient was placed in a supine position under general anesthesia, and an inflated tourniquet was used to stop bleeding in the affected limb. The incision was a median anterior knee incision with a medial parapatellar approach. The medial articular capsule was cut 1 cm from the medial edge of the patella to expose the knee joint.

For the gap balancing group, the distal femur was exposed without flipping the patella, then the femoral medullary cavity was opened. The control module was installed on the femoral aligner together with the distal osteotomy module. The femoral intramedullary positioning rod was inserted on the valgus angle control module. The active long nail on the aligner was inserted into the distal femur. The distal femoral osteotomy module was fixed with a fixed pin, and the osteotomy position was confirmed with an osteotomy measurement film. Then the intramedullary positioning rod was removed, and the distal femoral osteotomy was performed. An assembled tibial extramedullary positioning device was mounted on the lower leg, and a long nail at the upper end of the positioning rod was pinned into the proximal femur. The tibial osteotomy module was fixed with a fixed pin. After confirmation of the planimeter, the larger posterior osteophytes and residual meniscus were removed to obtain a balanced extension gap. The femoral dimension gauge was attached to the distal femoral osteotomy surface by hooking the posterior wings to the medial and lateral posterior condyles of the femur (using the default external rotation of 3 degrees as the rotation angle). The measuring hook on the femoral dimension gauge was moved to the highest point in the anterior cortex of the femur to determine the size of the osteotomy module based on the reader readings. Then the femoral dimension gauge was removed. The gravity method was used to re-determine the angle of lateral rotation osteotomy of the posterior femoral condyle. The assistant lifted the knee joint to allow it to sag naturally and used a matching G2 rectangular plate for scribe recording. Then the size of the femoral prosthesis was determined. The positioning holes of the osteotomy module were adjusted according to the scribes. The angle between the scribed line and the posterior condyle was measured. The positioning pin on the osteotomy module was inserted into the adjusted positioning hole. After a satisfactory flexion gap was obtained, femoral osteotomy was performed to obtain a good flexion gap, followed by measurement of the thickness of the posterior femoral condyle osteotomy.

For the measured resection group, except that the angle of lateral rotation osteotomy of the posterior femoral condyle was re-determined by the gravity method, the other steps were the same as that in the GB group. After measuring the angle between the horizontal line of the osteotomy plate and the connecting line of the posterior condyle, if there was an imbalance in the flexion gap, the surgical neck femoral epicondyle axis, and Whiteside line would be used to adjust the external rotation angle and then the osteotomy would be performed, and the soft tissue should be released to balance the extension and flexion gap. Then, the thickness of the posterior femoral condyle osteotomy was measured.

#### Prosthesis

The PFC Sigma fix platform prosthesis (Johnson, USA) was installed, then moved the knee joint and evaluated its stability and mobility to confirm the qualitative and patellar trajectory. The cement was used to fix the prosthesis.

#### Postoperative management

Postoperative drainage lasted 48 h until flow volume was normal and less than 30 ml. Ice was applied to the affected knee for 1 day to eliminate swelling. Rivaroxaban (10 mg/day) was orally administered for 21 days to prevent deep venous thrombosis in the lower extremities. Antibiotics were administered continuously for 3 days after the operation to prevent infection. All patients were treated with the same postoperative rehabilitation program. Two days after the operation, the patient began to walk down with a walking aid and had an active range of movement exercises at least 3 times a day for about 10 minutes each time.

### Data collection

Intraoperative indicators (operation time, osteotomy volume of medial, and lateral of posterior femoral condyles, external rotation angle) were recorded during operation. At 1, 3, 6, and 12 months after surgery, all the patients came to the hospital for review and underwent the following tests:

Pain severity: Pain severity of patients was assessed by visual analog scale (VAS) [11]. Total scores range from 0 to 10, with higher scores indicating greater pain.

Knee joint range of motion: The maximum range of angle that a patient can achieve in knee flexion.

Osteoarthritis index: The patients’ osteoarthritis index was evaluated using the Western Ontario and McMaster University Osteoarthritis Index (WOMAC) scale [12]. A total of 24 items including joint function, stiffness, pain, and other dimensions, with a full score of 96. Lower scores indicate better knee condition.

Squatting function: The seventh item of the Oxford Knee Score (OKS) [13] was used to evaluate knee squatting function in all patients. The OKS consists of 12 questions assessed by the Likert scale [14], with values ranging from 1 to 5 (1 = complete squatting, 2 = mild squatting difficulty, 3 = moderate squatting difficulty, 4 = severe squatting difficulty, and 5 = unable to complete squatting), and then the total score is calculated, of which 12 is the best score (least symptomatic) and 60 is the worst score (most symptomatic).

Knee function: American Knee Society Score (AKSS) [15] was used to determine the knee function of all patients. The AKSS includes walking distance (50 points) and ascending stairs (50 points). Higher scores indicate better recovery of knee function.

Follow-up: All patients were followed up for 1 year. WeChat and telephone were used to follow up with the patients once a week. The patients were required to follow up once a month in the outpatient clinic in our hospital. The occurrence of postoperative complications in all patients within 1 year was recorded in detail.

### Randomization and blinding

Randomization was performed by an independent researcher fellow using a number generated from a random number table, and the results were told to the surgeon the day before surgery. 96 patients were randomly divided into two groups: the gap balance group (*n* = 48) and the resection group (*n* = 48). The researchers, postoperative teams, and patients were blinded to surgical techniques until all clinical follow-up were completed and analyzed.

### Statistical analysis

All the data collected in this study were analyzed by SPSS23.0 software. Normally distributed measurement data were expressed as mean ± standard deviation (SD), while non-normally distributed measurement data were expressed as median (interquartile range), and the comparisons were examined by Student’s *t* test and Mann-Whitney test (non-parametric distribution). The categorical data were expressed as *n* (%), and the differences between the two groups were examined by chi-square analysis. *P*<0.05 was considered statistically significant.

## Results

**Fig. 1:**
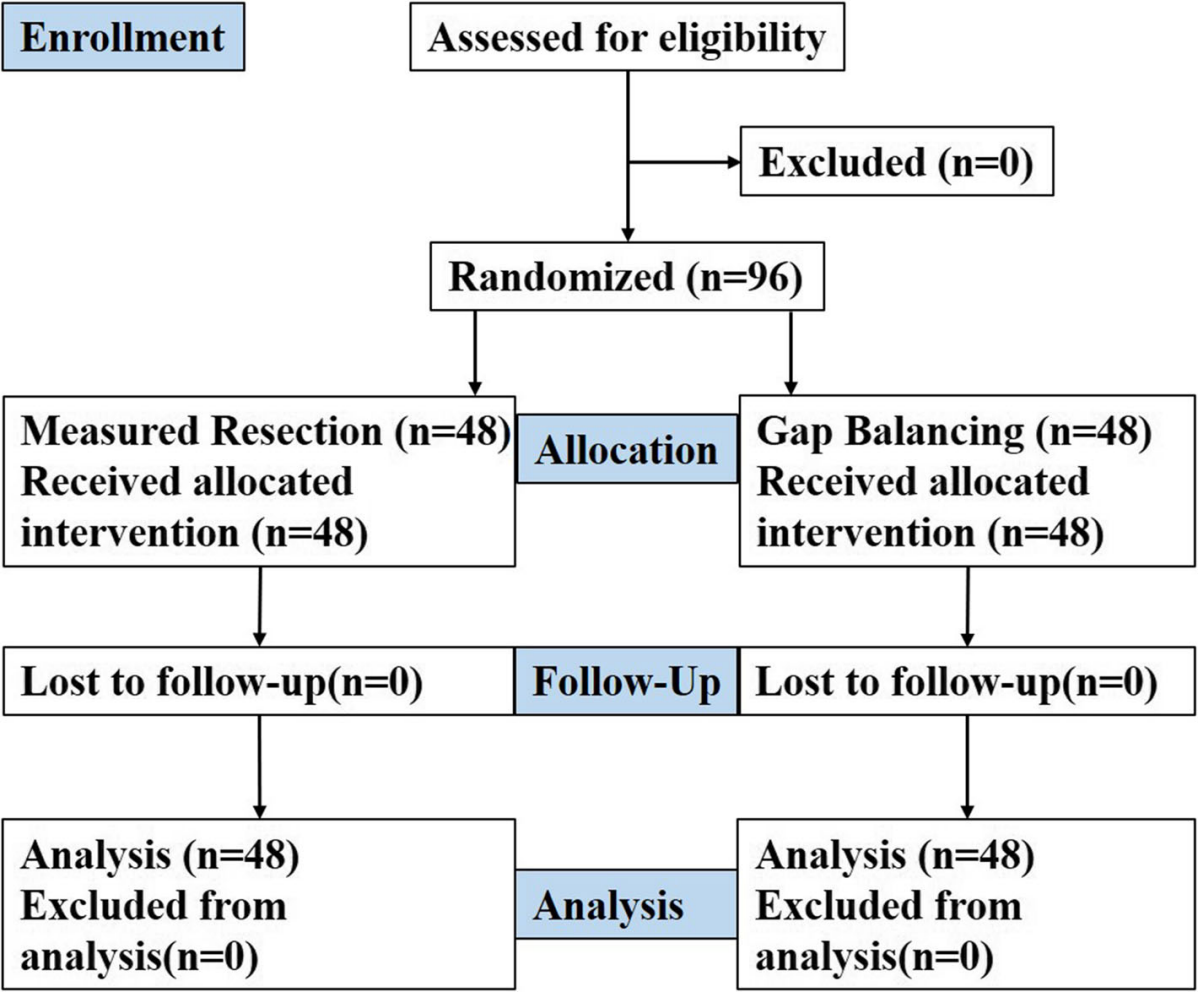
Flow chart for patient enrollment and randomization

Ninety-six patients who underwent unilateral primary TKA were eventually recruited for this prospective clinical trial. All patients received allocated intervention, and no patient was lost from the 1-year follow-up (Fig. 1). There were no significant differences in baseline patient demographics between the two groups in terms of age, sex, preoperative BMI, side of surgery, or American Knee Association score (*P* > 0.05) (Table 1).

**Table 1:** Clinical data of patients

Variable	Gap balancing *n* = 48	Measured resection *n* = 48	*P*
Age	60.29±3.16	60.18±3.28	0.868
Gender (M:F)	16/32	14/34	0.660
BMI (kg/m^2^)	22.09±1.63	21.93±1.92	0.661
side of surgery (left/right)	19/29	20/28	0.835
American Knee Association score	40.03±0.23	39.97±0.31	0.284

### Comparison of intraoperative indexes between the two groups

The osteotomy volume of the medial femoral condyle in the GB group was higher than that in the MR group (*P*<0.05), and the operation time in the GB group was shorter than that in the MR group (*P*<0.05). There were no significant differences in the osteotomy volume of the lateral posterior femoral condyle and external rotation angle between the two groups (*P* > 0.05) (Table 2).

**Table 2:** Intraoperative indexes of patients

Variable	Gap balancing *n* = 48	Measured resection *n* = 48	*P*
Operation time (min)	46.82±1.03	48.74±2.35	<0.001
Osteotomy volume of medial posterior femoral condyle (mm)	9.89±0.98	9.25±0.72	<0.001
Osteotomy volume of lateral Posterior femoral condyle (mm)	9.59±1.37	9.47±1.32	0.663
external rotation angle (°)	1.26±0.21	1.28±0.17	0.609

### Comparison of clinical outcomes between the two groups at 1, 3, 6, and 12 months after surgery

#### Pain severity

At 1, 3, 6, and 12 months after surgery, the pain severity in the GB group was lower than that in the MR group (*P*<0.05) (Table 3).

**Table 3:** Clinical outcomes

Variable	Time-point (months post-operative)	Gap balancing	Measured resection	*P*
Pain severity	1	2.48±0.07	2.87±0.08	<0.001
	3	2.09±0.13	2.42±0.09	<0.001
	6	1.68±0.15	2.12±0.17	<0.001
	12	1.52±0.09	1.91±0.11	<0.001
WOMAC	1	35.73±3.29	39.03±3.52	<0.001
	3	37.03±4.32	40.98±3.76	<0.001
	6	28.73±2.39	30.86±2.76	<0.001
	12	10.73±1.29	12.08±1.03	<0.001
Knee range of motion	1	107.93±7.63	101.39±6.38	<0.001
	3	108.83±6.91	102.93±5.51	<0.001
	6	109.23±6.63	103.28±4.39	<0.001
	12	109.45±6.29	103.65±3.29	<0.001
OKS	1	2.21±0.08	2.82±0.06	<0.001
	3	2.11±0.07	2.76±0.09	<0.001
	6	1.81±0.03	2.59±0.05	<0.001
	12	1.73±0.05	1.51±0.03	<0.001
KSS	1	82.02±1.98	79.87±1.02	<0.001
	3	84.93±1.73	81.09±1.23	<0.001
	6	85.37±1.69	82.21±1.34	<0.001
	12	86.08±1.73	82.87±1.45	<0.001

*WOMAC* Western Ontario and McMaster Universities Osteoarthritis Index, *OKS* Oxford Knee Score, *AKSS* American Knee Society Score

#### Knee range of motion

At 1, 3, 6, and 12 months after surgery, the knee range of motion in the GB group was larger than that in the MR group (*P*<0.05) (Table 3).

#### Western Ontario and McMaster Universities Osteoarthritis Index (WOMAC)

At 1, 3, 6, and 12 months after surgery, the WOMAC of the GB group was lower than that of the MR group (*P*<0.05) (Table 3).

#### Squatting function: OKS

At 1, 3, 6, and 12 months after surgery, the OKS of the GB group was higher than that of the MR group (*P*<0.05) (Table 3).

#### Knee function: AKSS

At 1, 3, 6, and 12 months after surgery, the AKSS of the GB group was higher than that of the MR group (*P*<0.05) (Table 3).

#### Complication

During the 1-year follow-up period, there were 7 cases of postoperative flexion instability and 2 cases of asymptomatic patellar bounce in the MR group. There was 1 case of knee flexion under-extension at 5 degrees and 1 case of anterior knee pain caused by patellar instability in the GB group. The incidence of postoperative complications in the GB group (4.17%) was significantly lower than that in the MR group (18.75%) (*P*<0.05).

## Discussion

A balanced and well-aligned TKA will provide a good functional outcome over the long term for patients taking a prosthetic TKA [16]. A 19-year follow-up of total knee arthroplasty showed that the utilization rate of prosthetic was 92.4% [17]. However, inappropriate intraoperative soft tissue release and osteotomy may lead to poor recovery of lower limb alignment and imbalance of flexion-extension gap [18]. Therefore, the method of intraoperative osteotomy and the accuracy of osteotomy have an important impact on the surgical effect and the prognosis level of patients. At present, TKA commonly used in clinics mainly includes MR technique and GB technique, but there are few studies on the effect of knee function and squat function in patients with knee osteoarthrosis after TKA. In this randomized controlled trial, we compared the effects of the MR technique and GB technique after surgery. We found that both techniques could significantly improve knee function and squat function and that the treatment effect of the GB technique was better than that of the MR technique.

MR technique is widely used in clinical practice, mainly through the posterior condylar plane 3degree external rotation line, intercondylar line and Whiteside line, and other bony anatomical landmarks to guide femoral rotation positioning [6]. The gap balancing technique relies on balancing the tension of the medial and lateral collateral ligaments to stretch the rectangular knee flexion gap so that the femoral external rotation osteotomy plane is parallel to the tibial osteotomy plane [19]. GB technique has advantages in patellar trajectory, flexion-extension stability, and femoral prosthesis rotation [20]. In this study, the osteotomy volume of the medial femoral condyle in the GB group was higher than that in the MR group, and the operation time in the GB group was shorter than that in the MR group, there were no significant differences in the osteotomy volume of the lateral posterior femoral condyle and external rotation angle between the two groups. The reason might be that the gap balancing method adjusts the flexion gap mainly by the amount of osteotomy after soft tissue balancing, which reduces the treatment of soft tissue and thus shortens the operation time. In the operation of the GB technique, adjusting the external rotation of the osteotomy plate is needed for most patients, which would result in different osteotomy volumes of the internal and external condyles, and the osteotomy volumes of the internal femoral condyle are significantly more than that of the external femoral condyle. However, the osteotomy volumes of the external femoral condyle are still controversial in current clinical practice. Cidambi et al. [21] showed that the osteotomy volumes of femur and tibia in GB technique were lower than that in MR technique. However, the results of Babazadeh [22] et al. suggested that the distal femoral osteotomy volumes of GB technique were more than that in MR technique.

Postoperative pain is one of the common complications after TKA, which can even affect the surgical treatment effect of patients with knee osteoarthritis. In this study, the pain level of knee osteoarthritis patients after receiving GB and MR techniques was measured and analyzed. The results showed that at 1, 3, 6, and 12 months after surgery, the pain level in the GB group was lower than that in the MR group. Which indicated that the GB technique was more effective in reducing postoperative pain.

Postoperative knee range of motion is one of the important indicators for evaluating the treatment effect of TKA. Accurate measurement of knee range of motion after TKA can not only provide guidance for patients to perform functional training, but also effectively help to prevent prosthesis loosening and wear, which is one of the important factors affecting prosthesis survival. Zhou et al. [23] reported that GB technique could improve the long-term knee mobility of patients. The results of this study showed that the knee range of motion in the GB group was higher than that in MR group at 1, 3, 6, and 12 months after the operation, which indicated that the GB technique can effectively improve the knee range of motion in patients with knee osteoarthritis after primary TKA.

The Chinese version of WOMAC translated according to WOMAC, a measurement tool developed and recommended by the American Rheumatoid Arthritis Clinical Research Group, has been widely used in the clinical evaluation of knee osteoarthritis in China. The scale can sensitively reflect the changes of symptoms in patients with knee osteoarthritis before and after treatment and has good sensitivity [24]. In this study, the WOMAC of the GB group was lower than that of the MR group at 1, 3, 6, and 12 months after surgery, which suggested that the GB technique could reduce the osteoarthritis index more effectively in patients taking primary TKA and help patients recover quickly in the early stage.

Total knee arthroplasty can improve patients’ squat function. A previous study that used an OKS to assess the squat function in patients undergoing knee surgery showed that the difficulty or inability to squat for patients after TKA was reduced, from 80% preoperatively to 39% 1 year after surgery, and the situation at 2 years after surgery was not significantly different compared with that at 1 year [25]. In our study, the OKS of the GB group was higher than that of the MR group at 1, 3, 6, and 12 months after surgery, which implied that the GB technique could effectively improve the squat function of patients with knee osteoarthritis after primary TKA, with rapid squat function improvement within 1 year. The reason might be that using the same distraction force to stretch the gap between the inside and outside of the knee in the flexion state in the GB formed a rectangular flexion gap, which was more accurate and individualized to install the prosthesis for each patient. Besides, the rectangular flexion gap prevented the posterior femoral condyle from warping when the patient stands up, which may be another important reason. In another study, knee squat function improved significantly better in the first year after TKA or unicondylar arthroplasty than that in the second year [26].

In this study, the AKSS of the GB group was higher than that of the MR group at 1, 3, 6, and 12 months after surgery. Consistent with previous studies in China, it suggested that the GB technique could effectively improve knee function in patients with knee osteoarthritis after primary total knee arthroplasty [27, 28]. The reason might be that the GB technique made the femoral axis of rotation closer to the anatomical perimeter with less damage to soft tissue.

We also found that the incidence of postoperative complications in the GB group (4.17%) was significantly lower than that in the MR group (18.75%), which suggested that the GB technique could effectively reduce complications in patients with knee osteoarthritis after primary TKA. Gap balancing tended to cause femoral prosthesis rotation in the clinic. In this study, one patient has a postoperative complication with anterior knee pain and another with knee flexion and under-extension of 5 degrees, these might be caused by femoral prosthesis rotation. Therefore, during the operation in GB, attention should be paid to correcting the angle between line A and line B marked by the two pinholes to made the angle was less than 2 degrees.

## Limitations

There are still some limitations to our study. The follow-up period of 1 year was relatively short. We emphasize that our findings apply only in the short and medium term. Long-term follow-up of this cohort may provide a more comprehensive understanding of the effect of the two surgical techniques on knee function and squat function. Our study was mainly conducted in our hospital, and it was a single-center study. The pain severity, knee range of motion, osteoarthritis index, squat function, and knee function were not examined before the operation, which limited the visual comparison of postoperative effect and preoperative condition.

## Conclusions

In conclusion, the GB technique can effectively shorten the operation time, relieve pain, improve knee range of motion, improve squat function and knee function, reduce osteoarthritis index, and reduce the occurrence of complications, which is worthy of clinical popularization and application.

## Data Availability

The datasets generated and analyzed during the current study are available from the corresponding author on reasonable request.
